# ORAL CONCURRENT SESSION 1: Equity, Public Health, and Policy

**DOI:** 10.1002/pmf2.12003

**Published:** 2025-01-05

Abstracts 9 – 18

THURSDAY

January 30, 2025

1:30 PM – 4:00 PM

Aurora Ballroom A

MODERATORS

Ebony B. Carter, MD

Aviva Lee‐Parritz, MD

